# Causes of stress and poor wellbeing among paramedic students in Saudi Arabia and the United Kingdom: a cross-cultural qualitative study

**DOI:** 10.1186/s12913-023-09374-y

**Published:** 2023-05-05

**Authors:** Adnan Alzahrani, Chris Keyworth, Caitlin Wilson, Judith Johnson

**Affiliations:** 1 School of Psychology, University of Leeds, Leeds, LS29JT UK; 2 Department of Basic Science, Prince Sultan bin Abdulaziz College for Emergency Medical Services, King Saud University, Riyadh, 11466 Saudi Arabia; 3 Yorkshire Ambulance Service NHS Trust, Wakefield, UK; 4 Bradford Institute for Health Research, Bradford Royal Infirmary, Bradford, UK; 5 School of Public Health and Community Medicine, University of New South Wales, Sydney, Australia

**Keywords:** Paramedicine student, College, Mental health, Wellbeing, Paramedic, Prehospital, University, Stress, Thematic analysis

## Abstract

**Background:**

Paramedicine presents students with numerous challenges, including factors threatening their wellbeing. Over the last two decades studies have illustrated that paramedics and paramedic students are more likely to have mental disorders than the general population. These findings suggest that course-related factors could be important in contributing to poorer mental health. However, few studies have examined factors related to stress in paramedic students, and none of these have included paramedic students from cross-culture. To address this, the present study (1) explored paramedicine students’ training experiences and other education-related factors that could affect their wellbeing, and (2) illustrated the possible differences and similarities between two cultures (Saudi Arabia and the UK) to determine whether the students’ cultural environment influenced factors related to their wellbeing.

**Methods:**

A qualitative exploratory research design was used. Twenty semi-structured interviews were conducted with paramedicine students from the United Kingdom and Kingdom of Saudi Arabia (ten participants from each country). Reflexive thematic analysis was employed as the analytical approach in this study.

**Results:**

Four major themes were identified which captured the contributors to paramedic students’ stress levels: (1) exposure to potentially traumatic events, (2) relationships and communication, illustrating the student’s personal and professional experiences with others, (3) programme atmosphere, demonstrating the challenges and support students encounter during their coursework and training, and (4) career, elucidating the pressure of future career expectations and predictions.

**Conclusion:**

The study revealed contributors to stress were similar in both countries. Better preparation can help to reduce the negative impacts of the possible traumatic events encountered on placements and supportive relationships, especially with proctors, can boost student wellbeing. Universities are able to address both these factors and help foster a positive environment for paramedicine students. As such, these results should help educators and policymakers when identifying and delivering interventions to support paramedic students.

## Introduction

Paramedicine is an integral component of the healthcare profession that specialises across a range of settings [i.e., emergency, and primary care; 1]. Despite its significance, a unified and worldwide definition of Paramedicine did not exist until Williams et al.’s [1] definition, which characterizes Paramedicine as a specialized field with multiple practice areas that play a crucial role in the healthcare profession. Globally, paramedics face numerous environmental challenges and practice-related hazards [2]. These stressors can significantly impact their physical and mental health [3–7]. Among paramedics, anxiety, depression, and post-traumatic stress disorder (PTSD) have been reported at a rate of 10–27%, which is higher than in the general population [8, 9]. Higher stress rates may occur prior to qualification when paramedics are still in training. Studies have indicated that the prevalence of anxiety amongst paramedic students is 24% [10], depression is 28% [8], and PTSD ranges between 16.8 and 20.2% [11]. A correlation was seen between higher mental distress levels among paramedic students and attitudes and engagement levels in training and the increased possibility of leaving the profession [12–15]. As a result, these challenges can pose a significant threat to paramedicine students’ course-related training [8] .

There are several possible factors which contribute to a higher risk of mental health problems in paramedic students. For example, paramedic courses can be particularly competitive for candidates to gain a place. In Saudi Arabia (SA), for example, the acceptance GPA for paramedic science can be higher than for nursing colleges and allied health profession courses (i.e., Occupational Therapy, Clinical nutrition, Clinical Laboratory Sciences), which is challenging and puts students under intense pressure [16]. Paramedic training and practice are also challenging as they often include exposure to highly stressful work environments [7]. Training often involves long hours, a known cause of stress in healthcare professional students [4, 17, 18]. Furthermore, while paramedic students experience exceptionally high levels of workplace stress, they may be underprepared to cope with them effectively [19, 20].

Cultural factors can also influence the causes of stress in paramedicine students. For example, in SA, these stressors are compounded by cultural stigmas related to mental health [21–23]. Conversely, ambulance personnel in the United Kingdom (UK) face various factors such as social, cultural, biological, and psychological related that affect their wellbeing and lead to high attrition rates among paramedics, such as pressures at work, salary, lack of support, and failure to separate work from personal life [24, 25].

Accordingly, the extant literature has emphasised the need to provide those who work in the prehospital setting (including paramedic students) with early mental health interventions and continued support [26]. However, while some studies have investigated paramedic students’ wellbeing within the context of individual Western countries, there have been few studies in non-Western nations or cross-cultural studies on this topic [20, 27]. Indeed, only two cross-cultural studies have compared the mental health of paramedics from Western and non-Western nations [28, 29], and these were on qualified paramedics rather than students. Furthermore, few studies have investigated factors contributing to stress in paramedicine students [8]. Thus, overall there is a need for research on paramedicine student wellbeing in non-Western cultures and cross-cultural research related to paramedicine student wellbeing.

To date, no qualitative study has examined paramedicine students’ mental wellbeing between different Western and non-Western cultures. Such an approach could generate a richer and more nuanced understanding of this phenomenon. Accordingly, this study sought to address the gaps in the literature by conducting a qualitative cross-cultural study on SA and UK paramedicine students. The main aim of this study was to explore the perceived causes of stress and poor mental health in paramedicine students from two countries, SA and the UK. Specific aims were to (1) explore SA and UK paramedicine students’ training experiences and other education-related factors that could affect their wellbeing, (2) illustrate the possible differences and similarities between the two cultures and explore whether the students’ cultural environment influenced factors related to their wellbeing.

## Methods

### Participants and procedure

Purposive sampling was used to recruit students currently enrolled in a paramedicine program in SA or UK. We sought to ensure equal representation across genders and countries (SA and UK) and to capture a range of universities and training experiences, given that these factors could be associated with different stressors and mental health experiences. Overall, our goal was to ensure that the dataset was inclusive and representative of the diverse experiences of paramedic students in both countries. The study aimed to recruit 20–30 undergraduate paramedic science students from universities in the UK and KSA. Program coordinators of universities that teach paramedicine in these countries were identified through each university’s official webpage and contacted via email. The email contained an information sheet, the informed consent form used for the study, and a Qualtrics and email link at which interested students could contact to participate in the study. The inclusion criteria for participants were: (1) being a paramedic student in a UK or Saudi university, and (2) currently enrolled in a paramedicine program. For students who agreed to participate, information was collected about their demographics (i.e., age, country of residence, ethnicity, gender, marital status) and paramedic studies (i.e., coursework, type of paramedic program, year of study, highest degree received, and employment status). The recruitment started in April 2022 for KSA participants and in May 2022 for the UK participants. In June 2022, we stopped the recruitment; the last interview was conducted on the first of August 2022. There were 20 participants (SA: n = 10; UK: n = 10) as we targeted to reach up to 20–40 participants, which according to Hagaman & Wutich [30], should be the targeted in ‘metathemes’ studies. Both groups were interviewed remotely, and the interview sessions were recorded and transcribed verbatim via Microsoft Teams [Version 1.6.00.1159; Microsoft Inc] by AA, which JJ and CK verified them.

### Design

This study utilised a qualitative exploratory research design via a one-to-one semi-structured interview approach. A qualitative paradigm was instrumental in this research, given its capacity to generate profound, nuanced findings. A semi-structured interview schedule was developed by the research team to examine participants’ beliefs and opinions regarding the impact of paramedic coursework and training on their wellbeing. This schedule directed the interview process as a topic guide (presented in Table 1) helping participants engage with the one-to-one format thus it is not validated. In addition, it facilitated discussion of participants’ experiences with sensitive topics relevant to the study aims, as they were not restricted or otherwise influenced by privacy-related concerns [31]. The interview schedule included questions on availability and preference of wellbeing interventions, but the present analysis focuses solely on causes of stress.

### Data collection

All interviews were conducted by AA in English language, remotely via a video platform between April and June 2022. In accordance with the purpose and goals of the analysis and the ‘information power’ concept in qualitative research, data collection was terminated, and recruitment was ceased after n = 20 (n = 10 for both groups [32, 33]. Data from all participants were included in the analysis, but following data analysis, the team agreed that SA = 8 and UK = 7 was the point at which no new themes were identified [32, 33].

### Data analysis

Reflexive thematic analysis was employed as the analytical approach in this study [34]. This method provides a more nuanced understanding of the topic by enabling detailed identification of data and themes, which enhances depth of interpretation [34]. Furthermore, the method is well-suited for gaining insight into participants’ perspectives. The analysis interpretations can inform research and policy development, thus benefiting participants and stakeholders [35]. To execute the approach properly, Braun and Clarke’s [34] six steps were followed. The first step required familiarity with the data; thus, full transcripts were read several times before any coding occurred by A.A while C.K. and J.J. double-checked 10% of all transcripts. A manual approach following an inductive analysis technique was utilised for coding, with A.A. creating the themes and J.J. and C.K. verifying them. An open approach was used for initial coding in the analysis stage to verify that the code and themes were grounded in the data through a meeting between the research team (A.A., C.K., J.J.). Further, the codes and themes were initiated without separation between participants from SA and UK.

Subsequently, semantic-level coding was used to examine ideas explicitly expressed by participants in both groups, ensuring that analysis was guided by theory to reach an interpretation of findings related to factors affecting students’ wellbeing. The codes were then grouped into themes, with each theme representing a concept underscored by a precise pattern in the data [36]. Finally, the analysed data were linked to the unique experiences of paramedic students from SA and UK.

### Reflexivity and researcher characteristics

The lead author for the paper (AA) is a Saudi paramedic researcher, and it was recognised by the authorship team that his personal background and experiences may have influenced his interpretation of the data. Being also a faculty member in Saudi Arabia and not a UK paramedic led his position to be that of both an insider and an outsider to certain parts of the dataset. The research team took measures to minimize potential bias by interviewing participants with diverse backgrounds and carefully considering their perspectives. To support triangulation of the data, two transcripts were reviewed independently each by JJ and CK and codes were discussed between AA, JJ and CK. Arising themes from the data were also discussed between AA and CW, a UK paramedic, to provide further feedback. These efforts aimed to prevent the lead researcher’s positionality from limiting their interpretation of the data.

### Ethical approval

The study received ethical approval from the Ethics Committee in the School of Psychology at the University of Leeds (Ethics Reference No: PSYC-528; 06/05/2022).

## Results

In total, 20 in-depth semi-structured interviews were conducted with 10 paramedic students from SA and 10 paramedic students from the UK, representing various governmental and private academic institutions.

Regarding participants from the SA, seven were male, and three were female, with a mode age of 20–25 (n = 10). All participants were Saudi nationals, single, and enrolled as undergraduate students seeking bachelor’s degrees. The participants’ years of study ranged from Year 3–5 as undergraduate paramedic programmes in SA consist of four years of study and one year of internship.

Regarding UK participants, three were male, and seven were female, with a mode age between 19 - >31. All participants were British nationals, with seven identifying as single, two as married, and one as divorced; all seven participants were undergraduate students seeking bachelor’s degrees, with three having already obtained associate degrees. The participants’ years of study ranged from Year 1–3 as undergraduate paramedic programmes in the UK consist of three years of combined study and training. Demographic details are provided in Table 2.

The interviews identified four major themes: (1) exposure to potentially traumatic events, (2) relationships and communication, illustrating the student’s personal and professional experiences with others, (3) programme atmosphere, demonstrating the challenges and support students encounter during their coursework and training, and (4) career, elucidating the pressure of future career expectations and predictions (see Table 3).

### Theme 1: exposure to potentially traumatic events

Participants from both cultures acknowledged that paramedicine left them vulnerable to traumatic events that could adversely impact their wellbeing, particularly during training. Subthemes include patients, training, and vulnerability.

#### Patients

Participants from both groups described traumatic events related to patient care (e.g., death). They further reported concealing these events from family and friends to protect their wellbeing. For many, their first clinical case was a major challenge, and it made a strong impression on them:

“Umm, you know, I can’t unsee the things I’ve seen. I can’t forget the things I know. But I can protect other people from seeing and learning about those things.“ [P5, UK]

Participants also experienced stress working with specific types of patients, such as communicable disease patients, due to the risk of infection and transfer to family. Some SA participants reported significant stress working with older patients, which could be linked to cultural concerns around respecting older people. Both SA and UK participants found it challenging to treat paediatric patients:

“I remember two cases. In my first two cases, the first case was that I took my shot for hepatitis, and then the first case was a patient with hepatitis. So, I was terrified at the start; I still worried that the infection might have transferred to me and my family. So, you stay stressed until you get home, shower, and change.“ [P2, SA]

UK participants who were also parents found it difficult to treat patients the same age as their own children. The death of young patients was traumatic for them and negatively affected their wellbeing. Furthermore, younger SA and UK participants found treating patients the same age as themselves challenging. Some participants discussed cases in which they knew the patient (e.g., family member, neighbour). Such cases negatively impacted their wellbeing as they were frequently reminded of them in everyday life:

“They were not immediate next-door neighbours ... She just wasn’t very well at all. I couldn’t put my finger on it. She’d been unwell and vomiting for two days ... and she died 2–3 hours later ... I think the more pressure was that I had to walk past their house every day.“ [P4, UK]

Participants from both cultures expressed mixed opinions regarding patient outcomes. Some reported seeking information about a patient’s outcomes and being unable to relax or sleep until they learned whether the patient was stable. Others stated that not knowing the outcome allowed them to believe everything possible had been done for the patient:

“I’ve lost my trust [after knowing the patient conditions]. You know, that was my first shift [in that week], I went to the 2nd shift and 3rd shift, and I wasn’t in a good mood, so I didn’t go to the 4th shift. Because it had got me to think maybe about this case 5–6 days after.“[P4, SA]

#### Training

All participants cited training environments as significantly impacting their wellbeing, and many identified them as a source of stress. Some had a negative impression when they looked to working paramedics for a glimpse of their future:

“I don’t want to have this negative feeling about my career and my place in the world. Yeah, that’s why I think [the work environment is] the worst thing.“ [P3, SA]

Training occurs in potentially challenging environments. Two primary sources of stress described by both groups were continuous training and working with new people in unfamiliar environments:

“It’s a challenge to work 12 hours with someone we don’t know, really. So, I mean, like the first month of my internship in *** prehospital. It was very difficult.“ [P1, SA].

Participants from both sides identified unsafe training environments as a significant source of stress, with several describing verbal or physical assault from patients’ family members or bystanders. Environmental challenges extended to cases where other healthcare providers overburdened the participants with tasks and emphasised their failures:

“She’s an Essex girl. She’s dumb. She’s, you know, all this, and I think just at some point just having to stand your ground. One of the crew bases I mentioned didn’t have a great attitude towards me, and he always says, you know, ‘Essex girls are like …’ whenever I start questioning a patient.“ [P6, UK]

In both countries, some female participants felt that the environment was challenging due to misogynistic sentiment, whether directed against them or other female medical professionals:

“They talked about a lady; she was conservative. She was wearing a hijab and everything, and then he is done advances with her [healthcare provider]. And then they did transfer her somewhere else. But then the whole staff, the doctors, the other nurses were saying it’s because she’s too conservative ... this environment is not for conservative people. They were blaming it on her, not on him.“ [P9, SA].

#### Vulnerability

Participants identified events where they felt vulnerable during training. Some described clinical placement training as the only factor that negatively affected their wellbeing as students, reflecting its significance as a source of challenge. For both groups, early clinical placements seemed to increase the risk of experiencing stress due to inexperience. Many paramedicine programmes, including those in the UK, provide students with training placements as early as the first year:

“I think going on placement so soon after maybe, like, six weeks of initial staff verify it was good and bad … However, the flip side also feels, like I mentioned, I didn’t know enough, if that makes sense. So, it was very overwhelming to then go on placement.“ [P2, UK]

Shift work was noted as a primary source of vulnerability as it can disrupt a student’s daily routine, sleep patterns, and overall wellbeing. Participants indicated that shift work could negatively affect their self-care, mental health, and social life:

“I think that—the changing of the shift time. Like in ***, they have two days and two nights. I think it’s affected my sleep.“ [P1, SA]

Some SA participants felt their presence during clinical placements was an obstacle to patient care, which negatively affected their wellbeing. However, UK participants did not echo this sentiment. Interestingly, UK participants identified initial patient interactions as a potentially stressful event due to their expectations in the face of uncertainty:

“So obviously, at first, it’s scary, and when we first started the ambulance service, I felt like there was a lot of responsibility on me, and I felt like I was underqualified to be taken on some of the challenges.“ [P3, UK]

### Theme 2: Relationships and Communication

Participants presented interpersonal relationships as a vital part of their life and mental wellbeing. They also felt that the development of their personality traits as students was affected by the relationships they maintained.

“I think the relationship, the personal relationships surely affect the well-being of the student; whether it’s a family relationship or even a relationship with friends.“ [P7, SA]

Many participants reported dividing their time between their college and training centre during clinical placement, reflecting the importance of relationships there. Subthemes related to relationships and communication include teaching faculty, peers and family, and training proctors.

#### Teaching faculty

Participants from both groups stated that faculty members significantly impacted their wellbeing. Some participants from both cultures reported that faculty mentors gave them advice and assistance to help them live healthier lives:

“Well, my support and encouragement were always there from our staff in the college; our doctors, our teachers, always encouraging us and telling us how we could bring this speciality up and we can lead this speciality [EMS] in the world and how we can always keep making it better.“ [P3, SA]

Conversely, participants from both cultures noted that faculty could negatively impact them through neglect. Thus, their relationships with faculty could either help or hinder their wellbeing:

“I think that can be challenging also on my course; I found that some people have more contact with the lecturers and … had better treatment of themselves. I’m not saying that affects their grade or anything, but I think those students get preferential treatment and might get certain things that other students don’t.“ [P8, UK]

Several UK and SA participants said they did not wish to burden faculty members. As students may not reach out when in need of support, teaching faculty should proactively establish an open dialogue with them. Students also valued the possibility of an open culture where emotions and coping strategies are freely shared:

“I find that quite frustrating, quite challenging because sometimes I find it hard to reach out to lecturers when I know they’re not treating everyone the same ... But I think because it is quite a high-pressure environment ... they might think that other students are absolutely fine. When that’s not the case, they just might not have the confidence to reach out.“ [P8, UK].

#### Peers and family

Participants cited support from peers and family as essential to their wellbeing. Both groups acknowledged the positive impact of social interaction at student clubs and events as frequent communication with peers could reduce stress. Indeed, both cultures identified peer-to-peer communication as a means of changing attitudes towards mental health:

“I saw that I worked with some friends of mine who are also burned out. Uh, I discussed it with them, and they agreed with me how this thing could be, this speciality. If you do not manage your stress well, you may be burned out more quickly than some other specialities. They were helping me to manage this.“ [P7, SA]

Participants from both cultures stressed that supportive peers were helpful while unsupportive peers were damaging, with positive communication amplifying positive effects and negative communication exacerbating negative effects.

“I was sitting with a few friends at college, and I talked about treating my mental health and stuff and general, it was really awkward. The looks on their faces, how they changed the subject like they don’t want actually to talk about that. I got the hint at the time and never talked about it because I think it’s uncomfortable for people.“ [P9, SA]

However, these negative effects could be mediated by communicating with others in the field.Additionally, participants from both cultures reported that family interaction could improve their confidence and stress management. However, they carefully moderated the information they shared with their family:

“Yes, they play a great factor in my wellbeing ... Sometimes it’s really critical cases, and I don’t want to tell them about it because it may be horrifying for them.“ [P3, SA]

#### Training proctors

The EMS team consists of two-to-three paramedics or emergency medical technicians (EMTs). Training proctors supervise clinical placements and act as a point of contact for paramedicine programmes. They are with paramedic students during their most impactful experiences. Therefore, their role is crucial to support students’ professional competence and psychological wellbeing. Participants described situations where they felt mentally and academically supported or harmed by their proctors:

“I think it’s bad, badly affecting me because there is no, like, a real preceptor in the field training. There is no someone to watch and support my learning … So, most of the people I work with them, in the field training, their objective is to treat patients.“ [P1, SA]

Participants identified three roles of the proctor: professional, friend, and mentor. When the proctor immediately adopted a clear role, especially as a friend and mentor, it made students feel welcome and reduced stress:

“Some colleagues [proctors] vary widely, especially in the field; one of them would like you just to stay quiet, and we are just here to do our jobs. The other one, he really doesn’t think it’s a job. He wants you to be just like his friend, and he wants to talk all the time. The third one. No, he wants to keep things professional.“ [P3, SA].

Participants from both cultures noted inadequate preparation for student-proctor interaction in their programmes. They advised that proctors should be carefully vetted, with incentives provided to encourage adaptation to the paramedicine programme:

“Have some kind of ... bonus or something that you know paramedics can elect to be mentors.“ [P1, UK]

Participants from both cultures identified their proctors as challenges or threats, especially during early interactions. Further, they felt that being in an unwelcoming environment was disturbing and harmful:

“He didn’t allow me to really talk to the patients ... I felt like I wasn’t wanted or welcomed. And it left me feeling really upset. So, I think if I had that mentor from the beginning, it would change my experience massively.“ [P2, UK]

Both groups identified communication during treatment processes as a major challenge. Multiple participants witnessed other paramedics committing errors or misconduct. Some responded by privately correcting or discussing the problematic behaviour. Others felt afraid or helpless to act, which was a significant source of stress. In some cases, participants were told not to intervene, even if the mistake was obvious:

“Mismanagement in the interventions of the case. Yes, actually, a lot because sometimes you cannot say anything. Because this is not your place, and sometimes you are afraid that you will be kicked off or change the place of the training.“ [P2, SA].

Participants suggested that proctors could help ease stress and the impact of trauma by communicating and empathising with students, and they were grateful to proctors who did so:

“They teach me how to deal with my emotions after the case ... They told me to engage more, to talk more, to do more.“ [P1, SA]

”Yeah, I think having a good mentor is so important to your training ... I’ve been, you know, as you’re waiting at hospitals and my mentors, he’ll sit down with me while we’re waiting to clear at the hospital, and he’ll talk through the case ... And what I did well, what I can do better.“ [P6, UK]

### Theme 3: programme atmosphere

Many participants reported spending most of their time at their respective colleges, reflecting the importance of the programme atmosphere as well as two subthemes: college experience and support.

#### College experience

There is considerable variety among tertiary academic programmes in SA and UK. Participants expressed strong views regarding the programmes in which they were enrolled. Some were pleased with their programmes and considered them well-designed. Others felt that their programmes were different from what had been presented, which negatively impacted their mental wellbeing. In SA, some programmes include a year of preparation for students entering health-related specialities. Unfortunately, participants felt the execution of these programmes created challenges and stress:

“On the programme, the first year before getting to college, it was the first year. It’s a common year. I think they call it [preparatory year]. It was a little stressful, and because I didn’t know what my speciality was, I didn’t know my destiny.“ [P1, SA].

Participants from both cultures reported experiencing stress due to an exclusive focus on scientific subject matter. They also felt that the predominantly academic nature of the programmes was unhelpful. While curricula from SA and UK programmes included mental health subjects, those with little to no focus on students’ wellbeing did not help participants with stress management:

“We do a presentation on how we deal with stress and our coping strategies, but they don’t actually teach you how to cope with stress or your strategies. They just ask you what yours are.“ [P6, UK]

#### Support

Cultivating open communication about mental health was cited as essential to prepare students to deal with current and future challenges. Participants from both cultures felt that academic institutions provided students with means of support, but most still perceived a general lack of attention to student wellbeing. Conversely, they observed that an isolated student could not seek support or discuss their negative experiences, thereby perpetuating those experiences. Some SA students highlighted the importance of extracurricular activities in their programmes, although UK participants did not echo this. They also noted the limitations of on-campus resources to support mental health and wellbeing. Both groups described significant challenges related to feeling unwelcomed or unsupported by their college or training centre:

“I’d say the support whilst I’m out on placement is the most stressful because, as I said, it can be quite isolating.“ [P8, UK]

One group of participants felt unsupported in dealing with issues related to their training and studies, including recommendations for solutions that did not meet their needs. Another group found support from paramedics or other professionals at the training centre. A final group found support from both their training centre and college, whether through direct or indirect interactions (e.g., online counselling):

“They do a tripartite review, involving my mentor, myself, and my personal tutor. They give us goals to meet and ask whether or not we need support and whether it be for wellbeing. They also send out lots of emails, I’d say. Every couple of weeks about wellbeing support.“ [P4, UK]

### Theme 4: Career

Participants indicated that their relationship with their speciality was important for their wellbeing, revealing two subthemes: (1) motivation to join paramedicine and (2) future career predictions.

#### Motivation to join paramedicine

Participants’ motivations and reasons for wanting to become paramedics varied. Lack of adequate knowledge was more apparent in SA participants and associated with poorer wellbeing. Furthermore, paramedicine was not the first choice for some SA participants, who had initially wanted to train in another health-related field (e.g., dentistry, general medicine):

“First, I did not choose the specific speciality. I chose medical health.“ [P1, SA]

UK participants were more aware of the nature of the speciality, which supported their wellbeing through preparation:

“I wanted paramedicine. Was definitely my top choice.“ [P2, UK]

Despite the connection between specialty awareness and overall wellbeing, participants with high awareness expressed significant anxiety regarding potential stressors:

“You can gain and learn a lot about it. It is a lot of fun. It has a lot of stress. I heard about that in the beginning, but I know I can, handle that at some level, so. Yeah, I was devastated at first, but when I joined the college, I really enjoyed it.“ [P3, SA]

#### Future career predictions

Discussion of participants’ current struggles facilitated diverse predictions about their future careers. SA participants were concerned about limited job availability, which harmed their wellbeing; UK participants did not share these concerns:

“Sometimes I worry about that, especially in the field I want to go in, but I always think I am young and can do it. I can, like, travel to some other places that need … crew ... It can be difficult. It can be hard, but I am young.“ [P3, SA]

Participants expressed concerns about their future work environments. Some UK and SA participants stated that their professional colleagues in hospitals did not always seem to appreciate them as members of the public do. Other SA participants proposed that hospital environments might be preferable to prehospital ones. They highlighted physicians for their perceived unprofessional behaviour towards paramedics, which was detrimental to the participants’ wellbeing. Alternatively, physicians were noted for their willingness to help paramedic students overcome challenges, which participants described as beneficial to their wellbeing:

“Because the doctor was great there, they taught me a lot of stuff. Like, they were treating us like a worker with them, you know, like a mate.“ [P5, SA]

Female SA participants expressed similar concerns to their male counterparts but with different perspectives. All were worried about job unavailability and work-related stress; however, female participants had added anxieties related to societal gender expectations:

“Just to be honest, for me, I mean this society ... That is, like, the most thing that I am worried about it.“ [P8, SA]

Female UK participants reported concerns regarding male-dominated specialities.

## Discussion

### Principal findings

This study explored the challenges SA, and UK paramedicine students encounter during their studies and training to identify factors affecting their wellbeing. Four major findings were identified: (1) the probability of exposure to traumatic events was a significant contributor to stress levels, which were mostly encountered during clinical placements; (2) challenges in personal and professional relationships and communication, which had the power to support but could also amplify stress when support was inadequate or unhelpful; (3) challenges related to the university programme atmosphere which is important as students spend the majority of their academic time on their campus; and (4) concerns related to the paramedicine speciality or a future career which were a significant cause of students’ stress and pressure linked to their future. Paramedicine programmes and clinical sites should acknowledge their potential to negatively impact students and implement better measures to support their psychological wellbeing.

### Consideration of previous research

Previous studies have shown that university paramedicine programmes and clinical training sites can significantly impact students’ wellbeing, for better or worse [37]. Although clinical training is essential to the paramedicine programme, it can be traumatic for students, especially in uncontrolled environments (e.g., prehospital; [38]). Our study extends this literature by showing that participants viewed training as a significant stressor, including the specific challenges of working with unfamiliar people, feeling unwelcomed by colleagues, and potential exposure to threats (e.g., the risk of assault). Such challenges affect the wellbeing of everyone involved [39]. Similar to Lawn et al. [2], our participants believed that patient care could be negatively affected by their limited knowledge and resources as students as well as other personal or patient-related factors (e.g., patient’s age).

Conversely, we found that training can motivate paramedicine students to become better healthcare providers, as reported in Holmes et al. [40] and Ross et al. [41]. According to the literature, preparation is essential to limit the negative effects of the challenges students encounter during training [40]. Additionally, our participants noted many issues they felt could be easily fixed, such as training implementation, early clinical placements, and training proctor-student communication. Unfortunately, some programmes may not have the resources necessary to provide students with such experiences and opportunities, particularly in rural areas [42].

Our study extends the literature by showing that university life as a major factor influencing their wellbeing, and interpersonal relationships are a major part of university life. However, students may experience challenges in their relationships with faculty, proctors, peers, and even family. Our findings show that cultivating and maintaining relationships within the university should be encouraged, as relationships with supportive faculty can help students cope with the challenges they encounter [43]. Participants noted that extracurricular activities also provided an outlet to deal with these challenges; complementing the findings of Almalki et al. [44] which illustrates the role of extracurricular activities as a powerful strategy for managing stress and bringing their life into balance. Regarding clinical training, our participants observed that their relationship with their training proctor could affect their wellbeing. Although McCall et al. [45] found that proctors can support paramedicine students’ wellbeing, Trede et al. [46] demonstrated that proctors can negatively affect students’ wellbeing. Training policies can further amplify this positive or negative effect.

Our findings suggest that the university paramedicine programme atmosphere and structure can also affect student wellbeing. Teaching styles and exam methods vary among programmes and can present students with significant challenges [47]. The present study’s participants expressed concerns regarding their programmes’ assessment methods and grading policies. These concerns could be addressed through monitoring methods designed to enhance students’ learning [48]. SA participants cited the preparatory year as one of the most significant challenges to their mental health, although the preparatory year is not universal among paramedicine programmes [49]. However, our findings suggest that the lack of consistency among university paramedicine programmes itself could present some limitations as it is burden on associated parties(e.g., training proctors). Regardless, the literature shows that students understand that, compared to other health specialities, paramedicine programmes are relatively new and still improving [50, 51].

Participants’ understanding of the paramedicine speciality and their future career predictions varied and that show how it could become a cause to stress. SA participants showed less awareness of their specialty than UK participants, resulting in unreasonable expectations regarding the challenges they would encounter. As Alshammari et al. [52] noted, prehospital is a challenging speciality. Our findings abouts career predictions from female participants are consistent with the extensive literature on gender roles in male-dominated specialities, including those in SA [53] with some great challenge linked to acceptance and ability to be productive and supported. Interventions at the university level could help students obtain greater awareness of their speciality before enrolling. Moreover, our participants mentioned the importance of communication within the speciality to effectively deal with challenges affecting their wellbeing, similar to Lentz et al. [54].

### Implications

This study is one of the first qualitative studies to explore the perceived cause to mental health problems, especially causes of stress in non-western paramedicine students. Our findings suggest that students need greater access to information about health-related specialities before choosing paramedicine. Universities can address this need by providing students with open and supportive learning environments. Specifically, SA universities could implement strategies to increase students’ awareness of paramedicine programmes as well as the challenges related to paramedicine training and practice.

Paramedicine university and training programmes must create an atmosphere where students feel cared about and supported, with faculty members and training proctors providing mentorship [55]. SA and more importantly UK universities can help improve paramedicine students’ wellbeing by providing them with inclusive social activities and projects that create a collaborative environment. They can also encourage students to build relationships with others in their programmes, including faculty. Similarly, SA and UK paramedicine training programmes can support communication between students and proctors as well as proctors and universities. In particularly challenging settings (e.g., prehospital), all potential threats should be prevented, if possible; otherwise, students should be provided with support (e.g., counselling/ mental health support, professional mentorship), both on-site and at their university.

Although paramedicine students encounter unique situations, the speciality is still critically neglected in research [56]. Additional research is needed to determine their needs and evaluate the effectiveness of the supports provided to them. Building upon our findings, future researchers could investigate the experiences of paramedicine students and factors that affect their wellbeing during their studies and training. They could also examine the impact of the identified challenges on students’ personal habits.

### Strengths and limitations

A strength of this study is the use of a diverse sample of paramedicine students from multiple universities in both SA and the UK. The use of purposive sampling facilitated the inclusion of different types of students (i.e., external and internal students), and a sufficient sample size was achieved. The analysis employed also featured many benefits, making it possible to identify important themes in the data and understand the relevant context.

However, our recommendations are exclusively related to outcomes concerning paramedicine students in SA and the UK. Additional factors affecting students’ wellbeing could be revealed by recruiting participants from other settings, as the systems and methods for teaching paramedicine vary by country. Future research could also implement alternative methodologies to understand further the challenges identified.

## Conclusion

This study revealed several challenges encountered at the university or in clinical training that influence the psychological wellbeing of paramedicine students. While the factors that affect students’ psychological wellbeing vary, the negative impact of the challenges that they face are preventable. Although university and training programmes aim to help rather than harm students, they must understand that some factors and challenges can have a negative impact on students’ wellbeing. Interpersonal relationships and communication also play a role. Studies investigating threats to student wellbeing and ways to proactively prevent them should be a priority. Consequently, paramedicine programmes directors and students can improve the future of patient care by taking steps to maintain students’ wellbeing and prevent the development of mental health disorders.

**Table 1:** The interview schedule

Section one (opening questions)	
	• Please tell me what made you choose paramedicine as a speciality?
	• Do you know if anyone has noticed that your mental health (e.g., moods, behaviour) has changed since becoming a paramedic student?
Section two	
	How have you found going on the clinical placements? – Explore initial impressions. – Explore learning experiences. – Overall, illustrate anything positive. – Explore any possibly problematic elements. – Explore the support/encouragement received during the training. _Explore barriers encountered/how these were overcome?
	= What do you think of the structure of your training? – Explore the university’s approach to training. – Number of sessions – were there too many? too few? – Length and time of the shift – were they long enough to cover the requirements – too long to be achievable – Explore the grading methods of the training.
	Which part of the training is challenging from your experience as a paramedic student? – Explore any stressful challenges. – Explore a personal experience. – Explore the causes of the challenge. – Explore means to overcome challenges.
	To what extent can the training environment be challenging? – Explore any part of the environment that is challenging. – Explore the cause of the challenge. – Explore the role of colleagues. – Explore recourses and guidance sources.
Section three	
	What factors from your experiences affect paramedic students’ wellbeing the most? – Explore an incident that presents a factor where the well-being was impacted. – Explore university factors – Explore paramedic course and training factors – Explore personal relations and family factors
	How have you found your paramedic program? – Explore the relevance of teaching. – Explore the level of interest in teaching. – Explore the impact of exams and assessments.
	From your experience, how do you think training in the field would be a factor to affect the paramedic student wellbeing? – Explore an impactful case(s) – Explore the impact of the environment – Explore the impact of knowledge and preparation – Explore the impact of pre/post emergency call routine – Explore management approaches in cases of misconduct.
	To what extent does your future career worry you? – Explore job availability – Explore threats and challenges
	From your experiences, what factors do you think cause psychological problems in the EMS field? – Explore impactful case(s) – Explore thoughts and feelings.
	Does the term ‘psychological issues’ relate at all to the EMS environment? – Is it discussed among students? – Can you explore the nature of the discussions? – Is it discussed in positive/negative/stigmatising forms? – Who has these discussions? Among students? Between students and faculty?
Recommendations	What are your recommendations for future paramedic students regarding mental health challenges and factors impacting their well-being?
	Do you want to add anything that relates to the interview?

**Table 2:** Demographic Information

Country	Sex	Age	Year (*n*)	Ethnicity (*n*)	Interview time (Total time/average)
SA	Female = 3 Male = 7	19–25 (participants = 10)	Year 1–2 (0) Year 3 (3) Year 4 (1) Internship (6)	Arab Saudi (10)	Ranged 56–110 (793/79 min)
UK	Female = 7 Male = 3	19–25 (participants = 3) 26–30 (participants = 4) > 31 (participants = 3)	Year 1 (3) Year 2 (4) Year 3 (3)	White British (10)	Ranged 54–109 (754/75 min)

**Table 3:** Themes, descriptions

Themes	Descriptions
Exposure to potentially traumatic events	The identified theme suggests that paramedic students may experience adverse impacts on their wellbeing due to possible traumatic events, which were related to (1) patients, (2) training, and (3) vulnerability. **1. Patients**2. participants from both cultures reported experiencing traumatic events related to patient care, including death, as well as patient age (e.g., paediatric patients) and experience level (e.g., first clinical case)0.4. 5. **2. Training** Various aspects of training were identified as contributing to adverse impacts on the paramedic students’ wellbeing. These included unsafe training environments and events that were deemed unsuitable for practice and training. **3. Vulnerability** Paramedic students identified events that made them feel vulnerable, including early training placements as first-year students and involvement in shift work.
Relationships and communication	The identified theme highlights the significance of relationships and communication between paramedic students, their teaching faculty, peers, family, and training proctors for the students’ mental wellbeing. It was described as an essential aspect of their lives. **1. Teaching faculty** The wellbeing of paramedic students was significantly influenced by faculty members, with the extent of the impact varying depending on the faculty member’s chosen role. **2. Peers and family** The support provided by peers and family is essential to the wellbeing of paramedic students. **3. Training proctors** Establishing effective communication and relationships with training proctors is crucial for supporting paramedic students’ professional competence and psychological wellbeing. While some proctors negatively impacted the students’ wellbeing, others played a role in identifying factors that negatively affected the students and improved their circumstances.
Programme atmosphere	The identified theme emphasizes the importance of the program atmosphere, with many participants reporting spending most of their time at their respective colleges. This highlighted two crucial components of the program: (1) college experience and (2) support. **1. College Experience** The identified subthemes recognize the diversity of tertiary academic programs in both countries, including differences in program structure, educational methods, and subject materials, particularly those related to mental health. Participants from both cultures shared their views on the paramedicine programs they enrolled in, highlighting various components that negatively impacted their wellbeing. **2. Support** The identified subthemes focus on the type and availability of support that paramedic students received, as well as instances where they felt unsupported.
Career	The identified theme explores the relationship between paramedic students and their specialty, emphasizing its importance for their wellbeing. This theme reveals two subthemes: (1) motivation to pursue paramedicine and (2) future career aspirations. **1. Motivation to join paramedicine** The identified subtheme sheds light on the participants’ motivations and reasons for choosing to pursue a career in paramedicine. **2. Future career predictions** The identified subtheme addresses the current challenges that paramedic students face and explores their predictions for their future careers.

## Data Availability

The datasets generated and/or analysed during the current study are not publicly available due to the potential for individuals to be identified from the stories they share, even though these are anonymised; but are available from the corresponding author on reasonable request.
